# Do Health Expenditures Converge Among ASEAN Countries?

**DOI:** 10.3389/fpubh.2021.699821

**Published:** 2021-09-10

**Authors:** Zheng-Zheng Li, Guangzhe Liu, Ran Tao, Oana-Ramona Lobont

**Affiliations:** ^1^Department of Economics, School of Economics, Qingdao University, Qingdao, China; ^2^Department of Graduate School, Graduate School, St. Paul University Philippines, Tuguegarao, Philippines; ^3^Qingdao Municipal Center for Disease Control and Preventation, Qingdao, China; ^4^Department of Finance, West University of Timisoara, Timisoara, Romania

**Keywords:** convergence, public health expenditures, private health expenditures, panel unit root test, sequential panel selection method, ASEAN

## Abstract

This paper aims to determine the existence of convergence in health expenditures among Association for South East Asian Nations (ASEAN) countries. Based on the SPSM procedure and panel KSS unit root test results, the public health expenditures (PUHE) in Indonesia, Lao PDR, Cambodia, the Philippines, and Myanmar are converging, while that of Brunei Darussalam, Malaysia, Vietnam, Singapore, and Thailand are diverging. In addition, the sequences of private health expenditures (PRHE) in ASEAN member states are stationary, which implies convergence. This finding is in accordance with Wagner's law, that is, as nations develop, they are forced to expand public expenditure. Specifically, countries with low levels of PUHE tend to catch up with the high health spending countries. This research has policy implications with regard to the convergence of health expenditure across countries. The government in low- and lower-middle income countries should raise PUHE to provide access to health services for those who are unaffordable individuals.

## Introduction

This paper strives to debate the convergence of public health expenditure (PUHE) and private health expenditure (PRHE) among the Association for South East Asian Nations (ASEAN). Enriched health conditions are of great importance for human well-being and sustainable development strategy in any country (1, 2). The government's spending on health will promote public health outcomes and health capital accumulation (3, 4). Therefore, many authorities have adopted policies to highlight medical and health expenditures (5). Convergence is regarded as the consequence of a process in which the structures of different groups resemble each other (6). Following this, all countries would converge to a common equilibrium level of income (7). Health service is considered as a normal good in economics, which should exhibit similar dynamics to income. If there is evidence of health expenditure convergence, this suggests that targets to raise spending on public health are feasible. Reductions in disparities in health expenditure per capita between countries are, thus, evidence that such policies have been successful (8, 9). It is important to know how health expenditure changes concerning growth in the Gross Domestic Production (GDP).

There is evidence of a converging pattern of increases in health expenditure between low- and high-income countries, however, there still exist inequalities across countries (10). Currently, although most Asian countries have some form of state-run pension or social security program for health expenditure, coverage varies greatly (11). Coverage in PUHE tends to be higher in countries with higher GDP per capita (12). On the contrary, PRHE in ASEAN countries is approximately double that of the world average. According to Rancic and Jakovljevic (13), a high proportion of private expenditure on health has been found in Bangladesh and the Philippines. This high proportion of PRHE is a major concern, as it will aggravate existing poverty and compromise the welfare of the wider population of the ASEAN region (14). Since the resources are scarce in developing countries, if the allocation of budget in the health sector increases, individual health expenditure is expected to reduce significantly. Therefore, PRHE in parallel with PUHE to render health services has become the predominant concern for developing countries (15).

With the increasing globalization, urbanization, industrialization, and energy consumption, many countries pay attention to environmental pollution, social protection, and health conditions in ASEAN countries (14, 16). Health expenditure can be affected by macroeconomic variables, such as per capita GDP (17), technological advancement (5), population aging (18), and environmental pollution (19), etc. Countries with a higher proportion of older population tend to have larger health expenditure (16). Accompanied with the pressure of population aging (such as Singapore, Thailand, Vietnam, Indonesia, and Myanmar), health expenditure is emphasized in the ASEAN region (7, 20). Convergence is likely to have occurred in the economies that are more similar in medical technology, consumer preferences, health-related policies, and the health care system (21). Since the establishment of the ASEAN economic community in 2015, the economic integration in this region aims to achieve a competitive and equitable single market and enhance the status in the global economy (22). Economic integration also promotes the medical and health industry and has become an important development goal. The response has been positive to the concept of Universal Health Coverage (UHC) proposed by the World Health Organization (WHO) to achieve the Millennium Development Goals (MDG) (22). The government in ASEAN countries actively promotes the medical and health industry and related policy planning. For instance, Malaysia has included the development of health ICT in the 11th Malaysia Plan from 2016 to 2020, while Thailand has announced the “2016–2020 eHealth strategy” to drive the health care business in 2016^1^. However, the health expenditure still varies in ASEAN countries because of their distinctive conditions. Hence, it is urgent to examine whether the health expenditure in ASEAN countries converges in the context of economic integration.

We contribute to the current literature in the following aspects. Firstly, we concentrate on the healthcare expenditure issues in ASEAN countries, which have been neglected in previous studies. On the one hand, these countries have similar economic development levels, technique progress, and problems associated with aging. On the other hand, they have heterogenous health policies, which reflect the market and social choices concerning the supply of services, remuneration of health care providers, and the institutional arrangements for the finance of the health industry. These factors attract our interest to highlight the convergence of health expenditure in this region. Additionally, we distinguish the health expenditure into public and private segments across developing countries to evaluate the equity of their health systems. Finally, taking potential structural changes into account, we improve the non-linear Sequential Panel Selection Method (SPSM) and panel Kapetanios et al. (23) (KSS) unit root techniques to overcome the low power in detecting mean reversion. Furthermore, to deal with the cross-section dependence in the panel, we approximate the bootstrap distribution and this was not done in the previous study which assumes the cross-section independence. The empirical results report that the sequences of PUHE in Indonesia, Lao PDR, Cambodia, the Philippines, and Myanmar are stable, which implies that the PUHE are convergent. The PRHE in ASEAN member states has converging patterns, which is consistent with Wagner's Law. That, is, as nations become more advanced, they are forced to become more regulatory and improved, thereby expanding the public expenditures. Specifically, Hitiris (24) proposes that nations with low levels of health expenditure will catch up with high health spending nations. Therefore, the government in low- and lower-middle-income countries should offer more health resources for the poor to promote human capital.

The remainder of the paper contains the following sections. Section Literature Review summarizes related literature about this topic. Section Wagner's Law in Health Expenditure constructs the theoretical foundation in the field of public expenditure. Section Methodology describes the Sequential Panel Selection technique and advanced panel unit root test method. Section Data introduces the data and section Empirical Results analyzes the empirical result. The last section gives conclusions and implications.

## Literature Review

The concept of convergence has attracted lots of attention in an economic context. Theoretical discussions about convergence are based on the catch-up effect (24), which states that the GDP of slow-growing economies tends to converge to the fast-growing economies. Garrett et al. (25) propose that welfare would be converging because government size and growth are determined by the demand of its citizens or a collection of citizens organized into special interest groups. Interest groups can increase the size of government by organizing members and applying political pressure more effectively than individual citizens. Swank (26) argues that the social-economic issues created by market integration will put pressure on the government to expand welfare expenditure in the globalization process. By contrast, neo-liberal economics think globalization has limited the policy-making choices by forcing them to prioritize international competitiveness in their fiscal and economic policies while cutting back welfare expenditure. Wagner (27) believes that with the development of nations, they are forced to become more improved and expand public expenditure, which is fundamentally consistent with Swank (26).

Empirical studies mainly focus on the convergence phenomenon in developed countries. In specific, Hitiris and Nixon (28) employ the panel of European Union (EU) members' data and reveal that health expenditure exhibits β-convergence. Specifically, both absolute and conditional β-convergence are evident in per capita healthcare expenditure whereas only absolute β-convergence is supported for health expenditure as a share of GDP. Most scholars use a battery of unit root test to approximate the convergence process in empirical research. Hofmarcher et al. (29) recognize that health expenditure convergence occurs since the establishment of the Monetary Union, while before that, there is an extending wide gap in the average health expenditure levels in EU countries. Pekkurnaz (18) investigates the convergence of health expenditure by panel unit root tests in the Organization of Economic Cooperation and Development (OECD) countries and suggests that almost one-fourth of the countries were found to be converging between 1980 and 2012. Payne et al. (30) also take the OECD countries as an example and the unit root test results support for per capita health expenditure convergence among most OECD countries, and converging to the USA (31). Musgrove et al. (32) think that as income rises there is a convergence in the healthcare spending represented by public expenditure and out-of-pocket spending in WHO member states. Clemente et al. (33) found several convergence groups and reveal the existence of different patterns of behavior and disparities in the Spanish health system. However, contradictory conclusions are also proved by Lau et al. (7), who provide evidence that cannot be rejected by the existence of unit root for the health expenditure of most EU member states, even after taking non-linearity into account. Panopoulou and Pantelidis (34) propose that divergence in the full panel, but groups of countries can converge to different equilibria by using the Phillips and Sul (35) panel convergence analysis. Montanari and Nelson (36) confirm that European healthcare systems are not particularly hit by retrenchment and that convergence is absent in public healthcare dimensions. The existence of a single pattern of behavior of personal health care expenditure across the US states is rejected by Clemente et al. (37).

Despite extensive literature on the topic, studies about the path of health expenditure among developing regions is limited. The co-integration approach was applied to investigate convergence in health expenditure among the Economic Community of West African states by Oyedele and Adebayo (38). The finding is that the divergence exists in health expenditures, which indicates that there are differences across countries in health expenditures. In other words, the spending on the health industry will converge to its steady-state for each country. Odhiambo et al. (39) give evidence of the absolute and conditional convergence of health expenditure on a panel of 41 Sub-Saharan Africa countries for the period 2000 to 2011. Zhang et al. (40) point out that the government health expenditure disparities among the provinces in China are narrowing during 2003–2007 and the supply-side variables contribute to the government health expenditure convergence by taking the widely applied α and β-convergence tests. In the context of ASEAN countries, Sagarik (20) examines the determinants of PUHE and finds that older population and economic openness are negatively correlated with government health expenditure. Rahman et al. (5) reveal that the increase in health expenditure can promote the performance of health outcomes and quality of life (41).

However, there are obvious shortcomings in the extant studies. Firstly, from the perspective of this research object, most of the literature about the convergence of health expenditure has been conducted in OECD countries and other developed regions, while few focus on this issue in fast-growing emerging economies, such as ASEAN countries, or consider that the economic community has stimulated the integration of these member countries. This, an examination of whether the existence of convergence of health expenditure in ASEAN is necessary. Furthermore, most studies concentrate on aggregated or government health expenditure, while few studies distinguish specific health expenditure. Finally, considering the research method, the test procedure in most previous studies included α- and β-convergence which is based on the restrictive assumptions that countries follow the same growth path due to common technology, similar preferences, policies, and the potential for growth. The traditional linear unit root tests have low power when structural changes exist (42) and when there are problems with cross-section dependence. As we know, the global financial crisis in 2008 and the establishment of the ASEAN economic community will shock the stability of the series, then lead to structural breaks. The panel-based unit root tests are joint tests of a unit root for all members of a panel and are incapable of determining the mix of the *I*(0) and *I*(1) series in a panel setting.

## Wagner's Law in Health Expenditure

As we mentioned in the literature review, Swank (26) and neo-liberal economics have opposite ideas about public welfare expenditure in the globalization process. However, Wagner (27) found a common trend in public expenditure, which increases constantly over time within a nation. Therefore, they outline that as the economy grows, the activities and functions of the government also increase, which is called Wagner's Law. The mechanism can be explained from three perspectives. First, with the development of the economy, the demands for infrastructure and social protection increase because of the increased division of labor that increases with industrialization. Thus, nations have to increase their role in terms of public, regulatory, and protective activity. Moreover, increased urbanization and population density would require more public expenditure on law and economic regulation, highlighting administrative, and protective functions. Hence, as nations become more advanced, the market forces nations to become more regulatory and improved, thereby expanding public expenditure (20).

It can be further inferred that as citizens have higher incomes, they demand more education, entertainment, more equitable distribution of wealth and income, and generally more public services. In the meanwhile, people would like to have better access to and availability of knowledge on medical advances, which drives them to demand more diagnostic services, screening and monitoring, surgical procedures, and medical resources to raise life expectancy (43). More advanced health and medical technologies are thus invented or introduced from developed countries to less developed regions. Investment in health can improve human capital, which thus increases productivity and finally contributes to economic growth (44). Hence, from this point of view, the authorities must ensure a healthy workforce with the help of technological advances. In addition, spending on the healthcare industry can promote income equality and eliminate poverty (45). However, individuals tend to increase PRHE if they do not receive adequate public health services. Ultimately, countries with low levels of PUHE tend to catch up with high health spending countries (24). Fundamentally, the catch-up effect complements the application of Wagner's Law in the field of public expenditure.

## Methodology

Since non-linearities generally exist in macroeconomic and financial time series, which leads to conventional unit root tests having low power to detect the mean-reverting tendency of the series (42). Ucar and Omay (46) propose a non-linear panel unit root test by combining the non-linear framework in Kapetanios et al. (23) (KSS) with the panel unit root testing procedure of Im et al. (47). Perron (48) argues that if there is a structural break, the power to reject a unit root will decrease. Meanwhile, structural breaks are ignored in the data generating process, with analysis accepting the null hypothesis of a unit root. Therefore, Chortareas and Kapetanios (49) highlight the SPSM, considering the Fourier function. We believe this method is the best way to test for stationarity health expenditure among 10 ASEAN countries.

According to Kapetanios et al. (23), the KSS unit root test can detect non-stationarity against a non-linear but globally stationary exponential smooth transition autoregressive (hereafter, ESTAR) process. The model can be given by


(1) $$\begin{array}{r} \Delta {HE}_{t}=\gamma HE_{t-1}\left[1-\exp\left(-\beta HE_{t-1}^{2}\right)\right]+\omega_{t} \end{array}$$

where *HE*_*t*_ refers to health expenditure, ω_*t*_ obeys the standard normal distribution of zero mean with constant variance, and β ≥ 0 governs the speed of transition in the ESTAR process. Under the null hypothesis, *HE*_*t*_ has a linear unit root, but follows a non-linear stationary ESTAR process under the alternative. Kapetanios et al. (23) used a first-order Taylor function approximation for $1-\exp\left(-\beta HE_{t-1}^{2}\right)$ under the null hypothesis β = 0, as shown in the following function:


(2) $$\begin{array}{r} \Delta {HE}_{t}=\delta +\rho H_{t-1}^{3}+\sum_{i=1}^{k}\beta_{i}{\Delta HE}_{t-i}+\omega_{t},\;t=1,2,\ldots ,T \end{array}$$

In this function, the null hypothesis is ρ = 0 (non-stationarity) and the alternative hypothesis are expressed as ρ < 0 (non-linear ESTAR stationarity). Then, Ucar and Omay (46) expand a non-linear panel unit root test based on Equation (1), that is:


(3) $$\begin{array}{r} \Delta {HE}_{i,t}=\gamma_{i}H_{i,\;t-1}\left[1-\exp\left(-\beta_{i}HE_{i,\;t-1}^{2}\right)\right]+\omega_{i,t} \end{array}$$

Similarly, we consider the first-order Taylor series with the ESTAR model around β_*i*_ = 0 for all *i* in Equation (3):


(4) $$\begin{array}{r} \Delta {HE}_{i,t}=\delta_{i}+\rho_{i}H_{i,t-1}^{3}+\sum_{j=1}^{k}\beta_{i,j}{\Delta HE}_{i,t-j}+\omega_{i,t} \end{array}$$

where ρ_*i*_ = γ_*i*_β_*i*_ and we can get the hypotheses as follows:


(5) $$\begin{array}{r} H_{0}:\;\rho_{i}=0,\text{for all }i\;\left(\text{linear non}-\text{stationarity}\right)\\ H_{1}:\;\rho_{i}<0,\text{for some }i\;\left(\text{non}-\text{linear stationarity}\right) \end{array}$$

Combining the Fourier function with Equation (4), we construct as:


(6) $$\begin{array}{r} \Delta {HE}_{i,t}=\delta_{i}+\rho_{i}H_{i,t-1}^{3}+\sum_{j=1}^{k1}\beta_{i,j}{\Delta HE}_{i,t-j}\\ +a_{i,1}\sin\left(\frac{2\pi kt}{T}\right)+{b_{i,1}\cos\left(\frac{2\pi kt}{T}\right)+\omega }_{i,t} \end{array}$$

where *t* = 1, 2, …, *T*, *k* represents the frequency selected for the approximation, ${\left[a_{i},b_{j}\right]}^{\prime }$ measures the amplitude and displacement of the frequency, and $\left[\sin\left(\frac{2\pi kt}{T}\right),\;\cos\left(\frac{2\pi kt}{T}\right)\right]$ is selected in that a Fourier expression is capable of approximating integrable functions to any desired degree of accuracy. If there is a structural break, it must contain one frequency component^2^. Gallant (50), Becker et al. (51), Enders and Lee (52), and Pascalau (53) provide evidence that a Fourier approximation can capture the detail of an unknown aperiodic function. Moreover, the grid-search technique is performed to find the optimal frequency. Ucar and Omay (46) further explain that the statistic (hereafter, OU statistic) particularly suits small N observations.

The SPSM procedure contains three steps: firstly, the panel KSS test with a Fourier function is applied to all series in the panel. If the null hypothesis of unit root cannot be rejected, the procedure is terminated, which means all the series in the panel are non-stationary. Otherwise, we proceed to the next step; that is, we remove the series with the minimum KSS value from the panel because it is regarded as stationary; finally, we repeat the first step for the remaining series, or stop the procedure if all the series are removed from the panel. After the above three steps, the stationary series and non-stationary series can be separated.

## Data

The annual data ranging from 2000 to 2018 for PUHE and PRHE per capita of 10 ASEAN member states^3^ is sourced from WHO database. Health expenditure per capita series for these countries are adjusted by 2011 purchasing power parity (PPP) US Dollar to avoid the issues of exchange rate and inflation. To obtain the convergence effect, the health expenditure of country relative to the average health expenditure per capita among ASEAN countries should be constructed as indicated below (18, 30):


(7) $$\begin{array}{r} \alpha_{it}=\ln\left[HE_{it}/{average\left(HE\right)}_{t}\right] \end{array}$$

where *i* denotes the country; *t* represents time; *ln* is the natural logarithm.

Association for South East Asian Nations has achieved notable economic outcomes in recent decades (54, 55), which indicate that the total GDP in ASEAN has grown more than five-fold from 2000 and become the fifth-largest economy worldwide (56). However, these countries have undergone tremendous demographic, political, economic, and sociocultural transitions along with rapid economic development (57). Brunei, Cambodia, Malaysia, Singapore, and Thailand present the negative growth of the economy with the shock of the global financial crisis in 2008. The economy has been gradually recovering from the crisis in ASEAN since 2011. Moving to 2015, the economic community has been built. Due to the expansion of the middle class and the improvement of health awareness, the corresponding medical policies have been issued successively (22). The entire population has been covered by social health insurance in Thailand. In the case of Malaysia, the entire population has access to public health services, whilst in Singapore, more than 90% of the individuals are served by the compulsory government-organized health insurance scheme (58). Health insurance is available to more than half of Indonesians, with an ambition to achieve national coverage of UHC by 2019. Although big progress has been acquired in using health equity funds in Cambodia, the coverage of health insurance is still low in Cambodia (24%) and Lao PDR (15%).

In the process of globalization, Southeast Asia is the most active region, with many migrant workers flowing both within the region and between ASEAN. This presents other potential health concerns (58). Most migrants, still need to pay for medical expenditure privately in Singapore and the Philippines (58). Indonesia consists of many islands, which results in diseconomies of scale in the provision of health services by the government. Thus, private involvement was more dominant than government provision in health expenditure before 2010 (41). As a result, the Indonesian population is often forced to rely on private medical services out of necessity. As a complement to the public health care system, the rapidly-growing private sector offers curative and rehabilitative services and is financed on a non-subsidized, fee-for-service basis in Malaysia (59).

**Figure 1:**
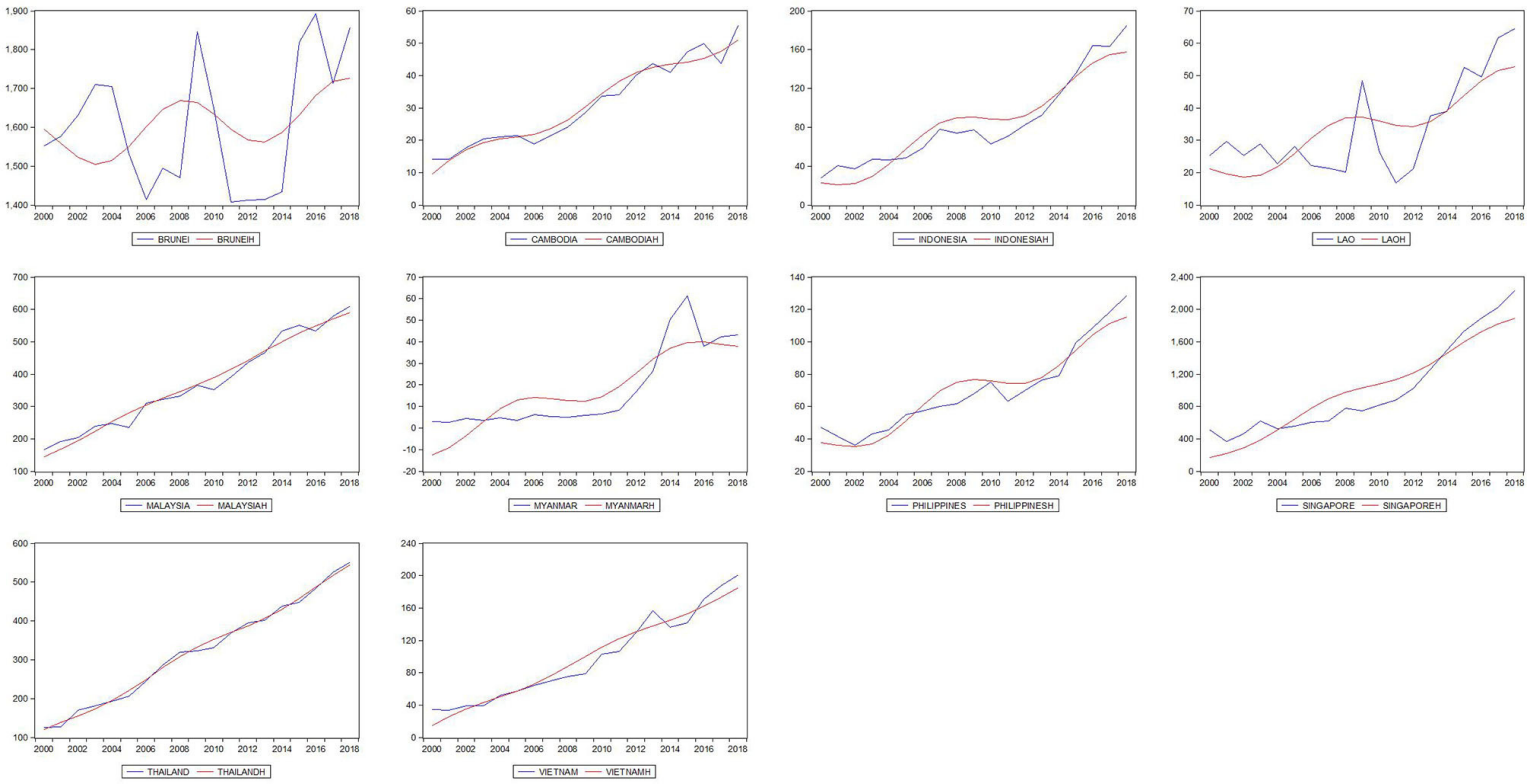
Plots of PUHE and fitted nonlinearities across ASEAN countries.

**Figure 2:**
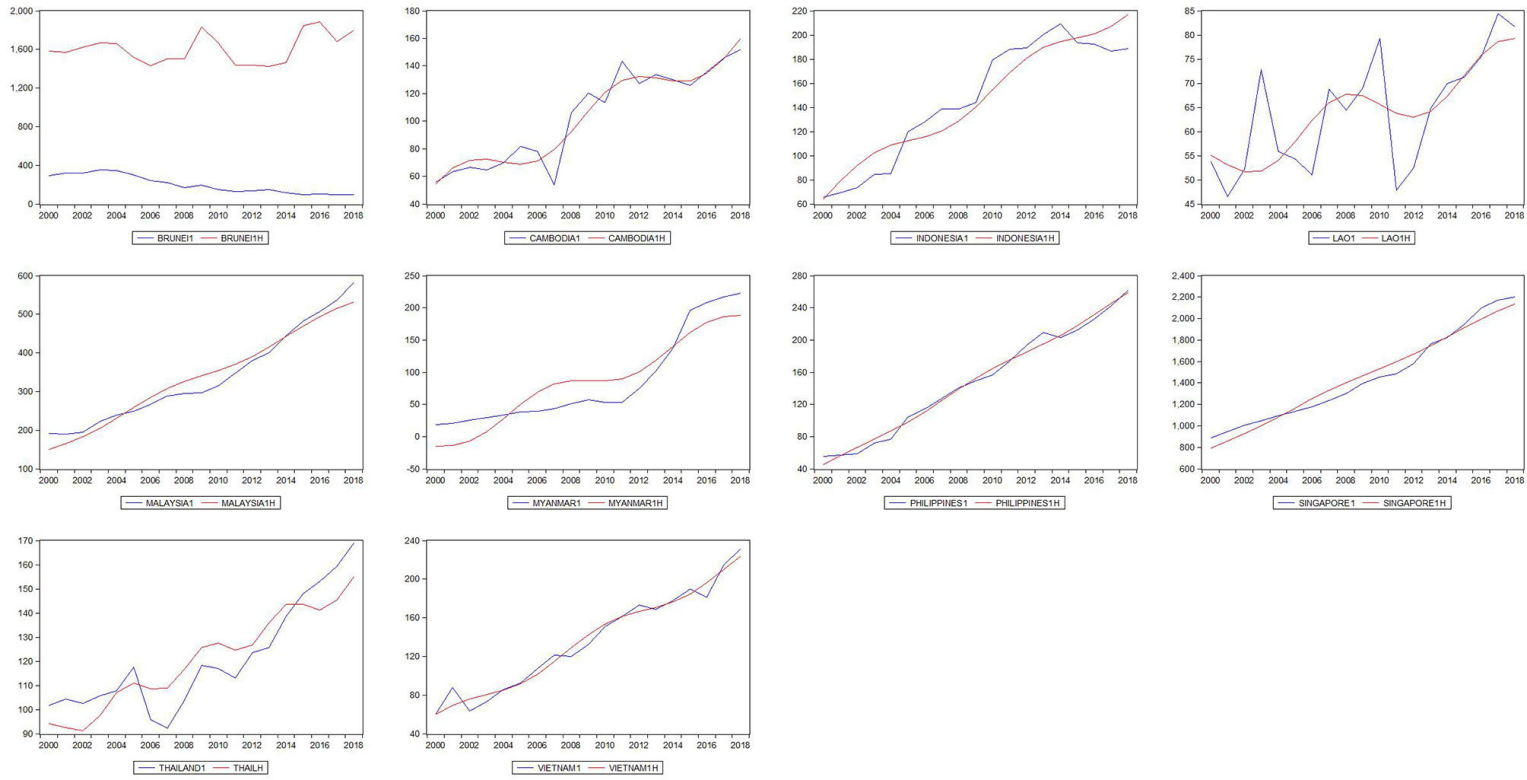
Plots of PRHE and fitted nonlinearities across ASEAN countries.

In Figures 1, 2 the deterministic trend plotted in red is closely fitted to the path of health expenditure at unknown breaking dates. Health expenditure in ASEAN countries shows fluctuating upward trends in the sample period. The PUHE shows a slight decline at the turning point of the global financial crisis in 2008, while PVHE keeps the increasing path during 2000–2018. The potential structural breaks in our dataset require a non-linear approach in the following trend analysis.

## Empirical Results

Table 1 lists the descriptive statistics of PUHE and PRHE in ASEAN members. It can be observed that there are huge differences in PUHE among ASEAN countries. Except for Brunei, Malaysia, and Thailand, the mean values of PUHEs exceed PRHEs, and the situation is opposite in other countries. Brunei has the highest mean PUHE while the lowest mean value is in Myanmar. Except for Singapore, there is little difference in PRHE among countries.

**Table 1:** Descriptive statistics.

	**PUHE**				**PRHE**			
	**Mean**	**Max**.	**Min**.	**Std. Dev**.	**Mean**	**Max**.	**Min**.	**Std. Dev**.
*Brunei Darussalam*	1606.875	1891.943	1407.643	167.290	200.708	351.669	94.878	94.047
*Cambodia*	31.076	55.556	14.046	13.116	103.479	151.962	53.754	34.440
*Indonesia*	84.590	185.061	27.633	46.778	146.019	209.340	65.603	50.486
*Lao PDR*	33.747	64.667	16.857	14.716	64.005	84.392	46.506	11.981
*Malaysia*	371.624	611.028	165.784	141.682	338.532	582.672	188.896	123.307
*Myanmar*	17.753	61.403	2.752	19.316	85.339	223.027	18.652	72.639
*Philippines*	70.214	128.622	36.115	26.575	149.194	262.291	55.215	67.165
*Singapore*	1007.087	2243.973	367.028	585.863	1461.443	2204.309	886.624	429.183
*Thailand*	322.323	551.219	125.161	133.306	120.878	169.057	92.354	22.496
*Vietnam*	98.715	200.541	33.384	54.496	136.449	231.499	60.447	51.763

The linear augmented Dickey-Fuller (ADF) (60), Phillips and Perron (PP) (61), and KPSS (23) unit root tests (with the trend and without trend) are applied to investigate the null hypothesis of a unit root in the PUHE and PRHE, respectively. The results for PUHE in Table 2 reveal clearly that the null hypothesis of a unit root without trend cannot be rejected except for in Thailand according to the PP test, while KPSS shows Indonesia and Lao PDR are stationary. In another case, we need to consider that there is a unit root for sample countries. Table 3 shows that the null hypothesis can be rejected for Cambodia and Thailand based on ADF and PP tests, while the KPSS test implies that Cambodia and Indonesia are stable. The results of unit root tests with the trend are basically in accordance with the above findings. However, structural breaks often exist in time series and are ignored by the linear unit root tests, which reduce the power of the linear unit root test (48). Moreover, the individual unit root tests do not consider the cross-section dependence across the ASEAN panel.

**Table 2:** Univariate unit root tests of the PUHE.

**Sequence**	**ADF**		**PP**		**KPSS**	
	**Without trend**	**With trend**	**Without trend**	**With trend**	**Without trend**	**With trend**
*Brunei Darussalam*	−2.120(0)	−3.220(0)	−2.233(1)	−3.215(2)	0.573[1]^**^	0.113[0]^*^
*Cambodia*	−0.015(3)	−3.984(3)	1.912(1)	−1.693(1)	0.466[3]^**^	0.121[2]^**^
*Indonesia*	1.579(0)	−3.248(2)	−1.604(1)	−3.038(1)	0.297[3]	0.155[2]^**^
*Lao PDR*	−0.962(0)	−2.892(0)	−0.843(0)	−2.741(3)	0.191[0]	0.160[2]^**^
*Malaysia*	0.167(0)	−1.857(0)	1.638(0)	−1.838(1)	0.449[3]^*^	0.174[2]^**^
*Myanmar*	−0.639(0)	−3.095(2)	−0.727(1)	−2.036(2)	0.505[3]^**^	0.133[2]^*^
*Philippines*	1.629(0)	−2.564(0)	2.073(3)	−2.655(1)	0.387[2]^*^	0.150[2]^**^
*Singapore*	−0.505(1)	−0.819(0)	0.361(1)	−0.781(2)	0.559[3]^**^	0.160[3]^**^
*Thailand*	−2.540(0)	−2.735(1)	−2.811(3)^*^	−2.604(4)	0.445[3]^*^	0.072[1]
*Vietnam*	−1.327(0)	−2.385(0)	−1.302(3)	−2.118(2)	0.532[3]^**^	0.186[1]^**^

* and ** *indicate significance at the 10 and 5% level, respectively*.

*The number in parenthesis suggests the lag order selected based on the recursive t-statistic by Perron (48). The number in the brackets indicates the truncation for the Bartlett Kernel proposed by the Newey-West (62) test (similarly hereinafter)*.

**Table 3:** Univariate unit root tests of the PRHE.

**Sequence**	**ADF**		**PP**		**KPSS**	
	**Without trend**	**With trend**	**Without trend**	**With trend**	**Without trend**	**With trend**
*Brunei Darussalam*	0.124(0)	−2.084(0)	0.202(2)	−2.296(2)	0.569[3]^**^	0.099[2]
*Cambodia*	−0.287(0)^*^	−3.044(0)	−2.857(1)^*^	−3.048(1)	0.278[2]	0.088[1]
*Indonesia*	−1.579(0)	−0.598(0)	−1.604(1)	−0.609(0)	0.297[3]	0.155[2]^**^
*Lao PDR*	−2.347(0)	−3.307(0)	−2.290(2)	−3.269(3)	0.573[2]^**^	0.079[2]
*Malaysia*	−0.403(2)	0.815(2)	0.719(1)	0.080(6)	0.562[3]^**^	0.161[3]^**^
*Myanmar*	−0.780(0)	−1.812(1)	−0.829(2)	−0.101(1)	0.505[3]^**^	0.146[3]^**^
*Philippines*	−1.518(0)	−3.553(2)^*^	−1.486(1)	−3.167(1)	0.491[3]^**^	0.057[1]
*Singapore*	−1.575(0)	−1.257(0)	−1.488(1)	−1.219(4)	0.525[3]^**^	0.155[3]^**^
*Thailand*	−2.125(0)	−1.293(0)	−3.282(1)^**^	−0.881(5)	0.514[3]^**^	0.180[2]^**^
*Vietnam*	−2.157(0)	−3.285(0)	−2.089(3)	−3.066(5)	0.460[3]^*^	0.188[3]^**^

* and ** *indicate significance at 10 and 5%, respectively*.

As the beginning, we apply the first-generation panel unit root methods by Levin, Lin, and Chu (LLC) (63). Im, Pesaran, and Shin (IPS) (47), and Maddala and Wu (MW) (64) to test the panels of PUHE and PRHE in Table 4. The results show that it cannot be rejected the null hypothesis of a unit root even under the 10% significance level. However, the first-generation panel unit root tests assume cross-sectional independence, which may not be applicable in a panel constructed by neighboring countries in the process of economic integration. In light of this consideration, the second-generation panel unit root tests by Bai and Ng (65), Choi (66), Moon and Perron (67), and Pesaran (68) are further employed. Table 4 provides evidence that PUHE and PRHE sequences contain a stationarity process under second generation panel unit root tests. Thus, the consideration of cross-sectional assumption gives more consistent results of convergence. However, the panel unit root tests cannot give the individual characteristics of certain economies. As we analyzed in methodology, the panel KSS unit root test with Fourier function not only takes account of structural breaks but also cross-section dependence, which allows individual nations to follow distinctive non-linear growth paths.

**Table 4:** Panel unit root tests.

	**PUHE**		**PRHE**	
**Type of test**	**Statistic**	***p*-value**	**Statistic**	***p*-value**
**FIRST GENERATION**				
LLC	0.482	0.685	1.157	0.876
IPS	−0.206	0.418	0.193	0.577
MW	23.065	0.286	19.609	0.483
**SECOND GENERATION**				
Bai & Ng	−0.974	0.835	−0.716	0.763
Moon Perron	−7.598^***^	0.000	−8.287^***^	0.000
Choi (P_m_)	1.651^**^	0.049	1.503^**^	0.066
Perron	−1.607	0.620	−2.235^*^	0.095

*, **, and *** *indicate significance at the 10, 5, and 1%, respectively*.

We then employ a panel KSS unit root test combined with the SPSM, under the non-linear framework with Fourier function. Fourier functions can capture any structural breaks of an unknown form as a smooth process (52). This test is also applicable for the existence of a cross-section dependence problem. The results shown in Tables 5, 6 are for 10,000 replication simulations.

**Table 5:** Results of panel KSS with Fourier test on PUHE.

**Sequence**	**OU statistic**	***p*-Value**	**Min KSS**	***k***	**Series**
1	−3.206^***^	0.000	−4.024	2	Indonesia
2	−2.882^***^	0.006	−2.736	2	Lao PDR
3	−2.736^**^	0.010	−2.574	2	Cambodia
4	−2.693^**^	0.026	−2.504	2	Philippines
5	−2.276^*^	0.091	−1.838	2	Myanmar
6	−2.088	0.172	−1.687	2	Brunei Darussalam
7	−2.441	0.152	−1.341	2	Malaysia
8	−2.612	0.140	−1.209	2	Vietnam
9	−2.327	0.174	−1.169	2	Singapore
10	−1.510	0.275	−0.304	2	Thailand

***, **, and * *indicate significance at the 1, 5, and 10% significant levels, respectively; k is the frequency; The asymptotic p-values are simulated by the Bootstrap method using 10,000 replications*.

**Table 6:** Results of panel KSS with Fourier test on PRHE.

**Sequence**	**OU statistic**	***p*-Value**	**Min KSS**	***k***	**Series**
1	−3.571^***^	0.000	−3.926	2	Cambodia
2	−3.408^***^	0.000	−2.870	2	Singapore
3	−3.392^***^	0.000	−2.835	2	Vietnam
4	−3.251^***^	0.001	−2.576	2	Lao PDR
5	−3.130^***^	0.002	−2.426	2	Thailand
6	−2.955^***^	0.005	−1.954	2	Malaysia
7	−2.514^**^	0.040	−1.947	2	Brunei Darussalam
8	−2.466^*^	0.093	−0.854	2	Myanmar
9	−2.984^*^	0.090	−0.845	2	Philippines
10	−2.297^*^	0.097	−0.276	2	Indonesia

***, **, and * *indicate significance at the 1, 5, and 10% significant levels, respectively; k is the frequency; The asymptotic p-values are simulated by the Bootstrap method using 10,000 replications*.

Table 5 indicates that after the panel KSS test, the null hypothesis was rejected at the 1% significant level with the statistic of −3.206. Therefore, we implemented the SPSM process and found the minimum KSS value in Indonesia, which reveals that the PUHE in Indonesia is stationary. We then removed this sequence from the entire panel and continued the first step for the remaining sequences. The statistics of −2.882 still imply we should reject the unit root at a 1% significance level. The corresponding series is Lao PDR, which has the lowest KSS based on SPSM, should be removed from the panel. This procedure continued until we could not reject the null hypothesis at the 10% significance level. We removed five sequences for Indonesia, Lao PDR, Cambodia, the Philippines, and Myanmar from these procedures, which indicates that only these five series are stationary. Similarly, the results shown in Table 6 reveal that all the series of PRHE can be rejected by the panel KSS unit root test, implying that PRHE in ASEAN countries is stable.

The government of the Philippines started the National Health Insurance Program (NHIP) to ensure financial risk protection for all citizens in 1995. All retirees from government and the private sectors, government employees, and their spouses, children, and retired parents are eligible for insurance coverage, and employer mandates ensure coverage for Filipinos working in the formal sector of the economy (69). Coupled with the UHC program, NHIP has covered 80% population, including the poorest 9.6 million families being subsidized by the national and local government. In 2013, the NHIP was amended in terms of the benefit design and provider payment mechanisms, and co-payments were reduced (58). In December 2010, the Philippines Department of Health decided to extend the NHIP to achieve UHC for all Filipinos (70). In the revised version, increasing support and reducing out-of-pocket payments became an important focus of health policy (71).

Indonesia highlights progress in terms of UHC and migrant protection because health expenditure can be regarded as an investment in human capital (72). Good human resource management used by Malaysia during the financial crisis has been proved to help the country overcome turmoil (73). In 2014, the government has aimed to achieve UHC by 2019 (58). Meanwhile, the authority implements the NHIP, named Jaminan Kesehatan Nasional (JKN). The JKN aims to integrate the three main existing fragmented schemes, that is, the government-financed health insurance program for the poor, Askes for civil servants and pensioners, and Jamsostek for formal sector workers. In addition to covering medical insurance, Askes and Jamsostek schemes also provide work-related injury, retirement, and death benefits. This health insurance program covers the workers in formal and informal sectors, and benefits for 60% of the population (74).

The Lao PDR is a low-income country where the poor and informal workers comprise 80% of the total population, private spending on health is more than 60% of total health expenditure (75). To provide more health services, financial protection, and generate resources for the health sector, the government intends to create a national health insurance authority to unify the four different social health protection schemes (76). In this program, the self-employed and families who live in poverty can be served and get subsidies. The expectation is that a unified institutional arrangement will lead to UHC by 2020. In addition, the Global Health Action proposed by the EU in 2015 is to strengthen the capacity of national institutes of public health in several low- and middle-income countries, including Lao PDR (77).

Cambodia, like many lower-middle-income countries, has committed to UHC and implemented reforms to expand the quality of services in the health system over recent decades (78). Policies include formal user fee exemptions, health equity funds sponsored by the government, vouchers, and health insurance (79). The funded target is especially for the poor, by removing financial barriers to public health facilities and reimbursement of health-related fees for the poor (80). Studies have confirmed the positive effect of public health services on the poor.

After the civil war and military regime, the Myanmar health system is a mix of public and private systems in terms of both financing and service provision. The Myanmar Ministry of Health started to repair the fragile health system in 2011 and increased health-related investment, setting the goal of achieving UHC by 2030. However, due to the lack of reliable medical insurance systems and the shortage of health financial funding, dependence on private funds is high, accounting for 80% of Myanmar's total health expenditure (81). As an important step to achieve universal medical coverage, the government increased medical expenditure by 8.7 times from 2011 to 2015.

The PUHE in the above countries shows the convergence attribution, while Brunei Darussalam, Malaysia, Vietnam, Singapore, and Thailand diverge. It can be found that the PUHE are relatively high in these economies, such as Brunei Darussalam and Malaysia, with an advanced economy and social development indicators. This result is in line with the study of Sagarik (20), who found that the Philippines, Cambodia, Indonesia, and Laos are in the bottom group in terms of public expenditure share of GDP. Healthcare for citizens and permanent residents is virtually free of charge. This supports the study by Lagomarsino et al. who believe many upper-income groups are progressing toward, or have already achieved UHC (82). On the contrary, the government in many low- and middle-income countries is striving toward universal coverage and trying its best to raise funds through various channels to expand welfare and health expenditure (83). On the other hand, Southeast Asia has huge population mobility and there are many migrant workers. The convergence in ASEAN countries confirms the compensation theory, which believes that globalization has a positive impact on public spending because of social and economic problems such as unemployment migrant and unequal income distribution. This will spur public welfare expenditure to help those who are negatively affected by globalization (26).

Based on the empirical results in Table 6, all the sample countries show convergence in PRHE. The backbone of health services is in the public sector, but the transition to a market economy has promoted the growth of the private health sector. In urban areas, private pharmacies and clinics are often the first choice for medical services (84). Urbanization can lead to expensive services because overcrowded medical facilities cause diseconomies of scale (85). The payment method of the medical market will also affect the difference in medical expenditure. Private or out-of-pocket systems are health payment schemes that fund health care (86, 87). Meanwhile, marketization brings diversified health products and promotes the prosperity of the health and medical industry. With the improvement of health awareness and the income level of nations, PRHE will increase accordingly.

## Conclusions and Implications

This paper explores the convergence of health expenditure in ASEAN countries by the SPSM and panel KSS unit root test method. The empirical results report that the PUHE in five economies (Indonesia, Lao PDR, Cambodia, the Philippines, and Myanmar) are stationary, which means they are converging. These countries are classified as low- or lower-middle-income countries, which are striving to increase PUHE to achieve UHC goals. While the PUHE in the other five ASEAN member states (Brunei Darussalam, Malaysia, Vietnam, Singapore, and Thailand) are diverging, they have already made certain achievements in the health industry. Additionally, the PRHE in ASEAN countries shows the convergence, although most of them are accepted to have the unit root by traditional unit root tests. The reason may be that marketization and globalization promote the prosperity of the health care industry, and leads to the diversification of PRHE. This finding supports Wagner's Law, which states that as the economy grows, the activities and functions of the government also tend to increase (27). In this context, economic development and urbanization would lead to an increase in PUHE, due to the increasing need for public health facilities. Consequently, countries with low levels of health expenditure may catch up with countries that have high healthcare spending.

According to the results, the following recommendations can be made: firstly, achieving convergence across countries can promote economic growth by encouraging countries to undertake expenditure on health care in a cost-effective way (18). The convergence in PRHE reflects the improvement of public health awareness and the high cost of medical technology. The government should assume more responsibility in offering PUHE for the poor, to whom health services may be unavailable. Furthermore, low- and lower-middle-income countries need to improve health expenditure to achieve the positive cycle of a labor-capital promoting economy and economic feedback of human capital (20). Finally, even though convergence in health expenditure has been confirmed in certain countries, governments should take into account the particular situation of that country, particularly the development process to implement appropriate health-related policies rather than copy the experiences of other countries (10, 36). As political will and health awareness increase, ASEAN has significant potential to become a force for better health services in this region. Ultimately, people can enjoy higher health standards, comprehensive social protection, and improved health status.

There are shortcomings to this study due to the limited availability of the sample period, and future research should therefore focus on the issues affecting healthcare or welfare expenditure in ASEAN countries to verify our conclusions.

## Data Availability Statement

The original contributions presented in the study are included in the article/supplementary material, further inquiries can be directed to the corresponding author/s.

## Author Contributions

Z-ZL: conceptualization, methodology, software, and writing. GL: data curation and original draft preparation. RT: visualization and investigation. O-RL: supervision and editing. All authors contributed to the article and approved the submitted version.

## Conflict of Interest

The authors declare that the research was conducted in the absence of any commercial or financial relationships that could be construed as a potential conflict of interest.

## Publisher's Note

All claims expressed in this article are solely those of the authors and do not necessarily represent those of their affiliated organizations, or those of the publisher, the editors and the reviewers. Any product that may be evaluated in this article, or claim that may be made by its manufacturer, is not guaranteed or endorsed by the publisher.

## Footnotes

^1^Sourced from: https://www.sohu.com/a/260435973_100091613

^2^Enders and Lee (52) suggest that the frequencies should be obtained according to the principle of minimization of the sum of squared residuals. However, their Monte Carlo experiments suggest that high frequency will leads to the loss of power, while one or two frequencies are appropriate.

^3^The ASEAN member states include Brunei Darussalam, Cambodia, Indonesia, Lao PDR, Malaysia, Myanmar, the Philippines, Singapore, Thailand, and Vietnam.

## References

[1] Romer D. Advanced Macroeconomics. New York: McGraw-Hill (2012).

[2] Razmi MJ Abbasian E Mohammadi S. Investigating the effect of government health expenditure on HDI in Iran. J Knowl Manage Econ Inform Technol. (2012) 2:1–8. Available online at: https://www.researchgate.net/profile/Seyed-Mohammad-Javad-Razmi/publication/340492299_Investigating_the_Effect_of_Government_Health_Expenditure_on_HDI_in_Iran/links/5e8cd01692851c2f52885c56/Investigating-the-Effect-of-Government-Health-Expenditure-on-HDI-in-Iran.pdf

[3] Becker GS. Health as human capital: synthesis and extensions. Oxf Econ Pap. (2007) 59:379–410. 10.1093/oep/gpm020

[4] Bloom DE Canning D. The health and wealth of nations. Science. (2000) 287:1207–9. 10.1126/science.287.5456.1207 10712155

[5] Rahman MM Khanam R Rahman M. Health care expenditure and health outcome nexus: new evidence from the SAARC-ASEAN region. Glob Health. (2018) 14:1–11. 10.1186/s12992-018-0430-1 30466452 PMC6249744

[6] Nixon J. Convergence of Health Care Spending and Health Outcomes in the European Union, 1960–95. University of York, Centre for Health Economics (2000). 10.7748/nm.8.2.39.s18

[7] Lau CKM Fung KWT Pugalis L. Is health care expenditure across Europe converging? Findings from the application of a nonlinear panel unit root test. Eur Bus Rev. (2014) 4:137–56. 10.1007/s40821-014-0014-9

[8] Bloom DE Canning D Sevilla J. The effect of health on economic growth: a production function approach. World Dev. (2004) 32:1–13. 10.1016/j.worlddev.2003.07.002

[9] Mishra V Smyth R. Convergence in energy consumption per capita among ASEAN countries. Energy Policy. (2014) 73:180–5. 10.1016/j.enpol.2014.06.006

[10] Eissa N. Pandemic preparedness and public health expenditure. Economies. (2020) 8:60. 10.3390/economies8030060

[11] Asher MG. Financing old age in Southeast Asia: an overview. Nanyang Wenti Yenchiu. (1996) 1996:72–98. 10.1355/seaa96e 12348604

[12] World Bank. Averting the Old Age Crisis: Policies to Protect and Promote Growth. Washington, DC: Oxford University Press (1994).

[13] Rancic N Jakovljevic MM. Long term health spending alongside population aging in N-11 emerging nations. East Eur Bus Econ J. (2016) 2:2–26. Available online at: http://eebej.eu/2016v2n1/2-26.pdf

[14] Lean HH Smyth R. CO_2_ emissions, electricity consumption and output in ASEAN. Appl Energy. (2010) 87:1858–64. 10.1016/j.apenergy.2010.02.003

[15] Hajibabaei H Sadeghi Soghdel H Faraji Dizaji S Ahmadi A. Health expenditures in developing countries: determinants and guidelines. J Res Health. (2020) 10:257–66. 10.32598/JRH.10.4.1411.1

[16] Sagarik D. Determinants of health expenditures in ASEAN region: theory and evidence. Millennial Asia. (2016) 7:1–19. 10.1177/0976399615624054

[17] Rana RH Alam K Gow J. Health expenditure and gross domestic product: causality analysis by income level. Int J Health Econ Manage. (2020) 20:55–77. 10.1007/s10754-019-09270-1 31313127

[18] Pekkurnaz D. Convergence of health expenditure in OECD countries: evidence from a nonlinear asymmetric heterogeneous panel unit root test. J Rev Glob Econ. (2015) 4:76–86. 10.6000/1929-7092.2015.04.07

[19] Haseeb M Kot S Hussain HI Jermsittiparsert K. Impact of economic growth, environmental pollution, and energy consumption on health expenditure and R&D expenditure of ASEAN countries. Energies. (2019) 12:3598. 10.3390/en12193598

[20] Sagarik D. (2014). Public expenditures on health in ASEAN member countries: an analysis of trends and policy determinants. In: International Conference on Trends in Economics, Humanities, and Management (ICTEHM'14). Pattaya.

[21] Wang Z. The determinants of health expenditures: evidence from US state-level data. Appl Econ. (2009) 41:429–35. 10.1080/00036840701704527

[22] Van Minh H Pocock NS Chaiyakunapruk N Chhorvann C Duc HA Hanvoravongchai P. Progress toward universal health coverage in ASEAN. Glob Health Action. (2014) 7:25856. 10.3402/gha.v7.25856 25476931 PMC4256544

[23] Kapetanios G Shin Y Snell A. Testing for a unit root in the nonlinear STAR framework. J Econ. (2003) 112:359–79. 10.1016/S0304-4076(02)00202-6

[24] Hitiris T. Health care expenditure and integration in the countries of the European Union. Appl Econ. (1997) 29:1–6. 10.1080/000368497327335

[25] Garrett TA Rhine RM. On the size and growth of government. Feder Reser Bank of St Louis Rev. (2006) 88:13–30. 10.20955/r.88.13-30

[26] Swank D. Global Capital, Political Institutions and Policy Change in Developed Welfare States. Cambridge: Cambridge University Press (2002). 10.1017/CBO9780511613371

[27] Wagner A. Three extracts on public finance. In: Musgrave RA Peacock AT, editors. Classics in the Theory of Public Finance. London: Palgrave Macmillan (1958). p. 1–15. 10.1007/978-1-349-23426-4_1

[28] Hitiris T Nixon J. Convergence of health care expenditure in the EU countries. Appl Econ Lett. (2001) 8:223–8. 10.1080/135048501750103890

[29] Hofmarcher MM Riedel M Röhrling G. Health expenditure in the EU: convergence by enlargement? IHS Health System Watch (2004) 1:2–7. Available online at: https://www.ihs.ac.at/departments/fin/HealthEcon/watch/hsw04_1e.pdf

[30] Payne JE Anderson S Lee J Cho MH. Do per capita health care expenditures converge among OECD countries? Evidence from unit root tests with level and trend-shifts. Appl Econ. (2015) 47:5600–13. 10.1080/00036846.2015.1054070

[31] Narayan PK. Do health expenditures ‘catch-up'? Evidence from OECD countries. Health Econ. (2007) 16:993–1008. 10.1002/hec.1196 17238220

[32] Musgrove P Zeramdini R Carrin G. Basic patterns in national health expenditure. Bull World Health Organ. (2002) 80:134–46. Available online at: https://www.scielosp.org/article/ssm/content/raw/?resource_ssm_path=/media/assets/bwho/v80n2/a09v80n2.pdf 11953792 PMC2567725

[33] Clemente J Lázaro-Alquézar A Montañés A. Convergence in Spanish Public health expenditure: has the decentralization process generated disparities? Health Policy. (2019) 123:503–7. 10.1016/j.healthpol.2019.03.003 30910477

[34] Panopoulou E Pantelidis T. Convergence in per capita health expenditures and health outcomes in the OECD countries. Appl Econ. (2012) 44:3909–20. 10.1080/00036846.2011.583222

[35] Phillips PC Sul D. Transition modeling and econometric convergence tests. Econometrica. (2007) 75:1771–855. 10.1111/j.1468-0262.2007.00811.x

[36] Montanari I Nelson K. Social service decline and convergence: how does healthcare fare? J Eur Soc Policy. (2013) 23:102–16. 10.1177/0958928712456574

[37] Clemente J Lázaro-Alquézar A Montañés AUS. State health expenditure convergence: a revisited analysis. Econ Model. (2019) 83:210–20. 10.1016/j.econmod.2019.02.011

[38] Oyedele O Adebayo A. Convergence of health expenditure and health outcomes in ecowas countries. Int J Econ Fin Manage. (2015) 4:46–53. Available online at: https://www.ejournalofbusiness.org/archive/vol4no2/vol4no2_1.pdf

[39] Odhiambo SA Wambugu A Kiriti-Ng Ang AT. Convergence of health expenditure in Sub-Saharan Africa: evidence from a dynamic panel. J Econ Sustain Dev. (2015). 6:185–205. Available online at: http://erepository.uonbi.ac.ke/bitstream/handle/11295/87793/Wambugu_Convergence%20of%20Health%20Expenditure%20in%20Sub-Saharan%20Africa.pdf?sequence=1&isAllowed=y

[40] Zhang G Zhang L Wu S Xia X Lu L. The convergence of Chinese county government health expenditures: capitation and contribution. BMC Health Serv Res. (2016) 16:408. 10.1186/s12913-016-1635-8 27538780 PMC4991013

[41] Rao RR Jani R Sanjivee P. Health, quality of life and GDP: an ASEAN experience. Asian Soc Sci. (2008) 4:70–6. 10.5539/ass.v4n4p70

[42] Chortareas GE Kapetanios G Shin Y. Nonlinear mean reversion in real exchange rates. Econ Lett. (2002) 77:411–7. 10.1016/S0165-1765(02)00157-X

[43] Simoens S Villeneuve M Hurst J. Tackling nurse shortages in OECD countries. (2005).

[44] Webber D. J. Policies to stimulate growth: should we invest in health or education? Appl Econ. (2002) 34:1633–43. 10.1080/00036840110115109

[45] Barro RJ. Economic growth in a cross section of countries. Q J Econ. (1991) 106:407–43. 10.2307/2937943

[46] Ucar N Omay T. Testing for unit root in nonlinear heterogeneous panels. Econ Lett. (2009) 104:5–8. 10.1016/j.econlet.2009.03.018

[47] Im KS Pesaran MH Shin Y. Testing for unit roots in heterogeneous panels. J Econ. (2003) 115:53–74. 10.1016/S0304-4076(03)00092-7

[48] Perron P. The great crash, the oil price shock, and the unit root hypothesis. Econometrica. (1989) 57:1361–401. 10.2307/1913712

[49] Chortareas G Kapetanios G. Getting PPP right: identifying mean-reverting real exchange rates in panels. J Bank Fin. (2009) 33:390–404. 10.1016/j.jbankfin.2008.08.010

[50] Gallant AR. On the bias in flexible functional forms and an essentially unbiased form: the fourier flexible form. J Econ. (1981) 15:211–45. 10.1016/0304-4076(81)90115-9

[51] Becker R Enders W Hurn S. A general test for time dependence in parameters. J Appl Econ. (2004) 19:899–906. 10.1002/jae.751

[52] Enders W Lee J. A unit root test using a Fourier series to approximate smooth breaks. Oxf Bull Econ Stat. (2012) 74:574–99. 10.1111/j.1468-0084.2011.00662.x

[53] Pascalau R. Unit root tests with smooth breaks: an application to the Nelson–Plosser data set. Appl Econ Lett. (2010) 17:565–70. 10.1080/13504850802112245

[54] Narine S. Forty years of ASEAN: a historical review. Pac Rev. (2008) 21:411–29. 10.1080/09512740802294689

[55] Mahbubani K Sng J. The ASEAN Miracle: A Catalyst for Peace. Kent Ridge: NUS Press (2017). 10.2307/j.ctv1xz0m3

[56] Yeh M Chu HP Sher PJ Chiu YC. R&D intensity, firm performance and the identification of the threshold: fresh evidence from the panel threshold regression model. Appl Econ. (2010) 42:389–401. 10.1080/00036840701604487

[57] Chongsuvivatwong V Phua KH Yap MT Pocock NS Hashim JH Chhem R. Health and health-care systems in southeast Asia: diversity and transitions. Lancet. (2011) 377:429–37. 10.1016/S0140-6736(10)61507-3 21269685 PMC7159068

[58] Guinto RLLR Curran UZ Suphanchaimat R Pocock NS. Universal health coverage in ‘One ASEAN': are migrants included? Glob Health Action. (2015) 8:25749. 10.3402/gha.v8.25749 25626624 PMC4308585

[59] Dahlui M. Aziz NA. Developing health service hub in ASEAN and Asia region country report on healthcare service industry in Malaysia. In: Tullao TS Lim HH, editors. Developing ASEAN Economic Community (AEC) into A Global Services Hub, ERIA Research Project Report 2011-1. Jakarta: ERIA (2012). p.65–110.

[60] Dickey DA Fuller WA. Likelihood ratio statistics for autoregressive time series with a unit root. Econometrica. (1981) 49:1057–72. 10.2307/1912517

[61] Phillips PC Perron P. Testing for a unit root in time series regression. Biometrika. (1988) 75:335–46. 10.1093/biomet/75.2.335

[62] Newey WK West KD. Hypothesis testing with efficient method of moments estimation. Int Econ Rev. (1987) 28:777–87. 10.2307/2526578

[63] Levin A Lin CF Chu CSJ. Unit root tests in panel data: asymptotic and finite-sample properties. J Econ. (2002) 108:1–24. 10.1016/S0304-4076(01)00098-7

[64] Maddala GS Wu S. A comparative study of unit root tests with panel data and a new simple test. Oxf Bull Econ Stat. (1999) 61:631–52. 10.1111/1468-0084.61.s1.13

[65] Bai J Ng S. A panic attack on unit roots and cointegration. Econometrica. (2004) 72:1127–77. 10.1111/j.1468-0262.2004.00528.x

[66] Moon HR Perron B. Testing for a unit root in panels with dynamic factors. J Econ. (2004) 122:81–126. 10.1016/j.jeconom.2003.10.020

[67] Choi I. Unit root tests for panel data. J Int Money Fin. (2001) 20:249–72. 10.1016/S0261-5606(00)00048-6

[68] Pesaran MH. A simple panel unit root test in the presence of cross-section dependence. J Appl Econ. (2007) 22:265–312. 10.1002/jae.951

[69] Kozhimannil KB Valera MR Adams AS Ross-Degnan D. The population-level impacts of a national health insurance program and franchise midwife clinics on achievement of prenatal and delivery care standards in the Philippines. Health Policy. (2009) 92:55–64. 10.1016/j.healthpol.2009.02.009 19327862 PMC2719679

[70] Tobe M Stickley A. del Rosario RB Jr, Shibuya K. Out-of-pocket medical expenses for inpatient care among beneficiaries of the National Health Insurance Program in the Philippines. Health Policy Plann. (2013) 28:536–48. 10.1093/heapol/czs092 23048125

[71] Obermann K Jowett M Kwon S. The role of national health insurance for achieving UHC in the Philippines: a mixed methods analysis. Glob Health Action. (2018) 11:1483638. 10.1080/16549716.2018.1483638 29914319 PMC6008596

[72] Grossman M. On the concept of health capital and the demand for health. J Polit Econ. (1972) 80:223–55. 10.1086/259880

[73] Smith W Abdullah A. The impact of the asian financial crisis on human resource management in Malaysia. Asia Pac Bus Rev. (2004) 10:402–21. 10.1080/136023804200026444

[74] Suryahadi A. Febriany V, Yumna A. Expanding social security in Indonesia: the current processes and challenges. In: Yi I, editor. Towards Universal Health Care in Emerging Economies. London: Palgrave Macmillan UK (2017). p. 373–403. 10.1057/978-1-137-53377-7_14

[75] Ahmed S Annear PL Phonvisay B Phommavong C de Oliveira Cruz V Hammerich A. Institutional design and organizational practice for universal coverage in lesser-developed countries: challenges facing the Lao PDR. Soc Sci Med. (2013) 96:250–7. 10.1016/j.socscimed.2013.01.019 23433544

[76] Alkenbrack S Jacobs B Lindelow M. Achieving universal health coverage through voluntary insurance: what can we learn from the experience of Lao PDR? BMC Health Serv Res. (2013) 13:521. 10.1186/1472-6963-13-521 24344925 PMC3893613

[77] Bromage I Wright EP Kounnavong S Sychareun V Venroij L. Research provides evidence for health policy in Lao PDR. Glob Health Action. (2020) 13:1791415. 10.1080/16549716.2020.1791415 32741351 PMC7480484

[78] Wiseman V Asante A Ir P Limwattananon S Jacobs B Liverani M. System-wide analysis of health financing equity in Cambodia: a study protocol. BMJ Glob Health. (2017) 2:e000153. 10.1136/bmjgh-2016-000153 28589000 PMC5321386

[79] Dingle A Powell-Jackson T Goodman C. A decade of improvements in equity of access to reproductive and maternal health services in Cambodia, 2000–2010. Int J Equity Health. (2013) 12:1–12. 10.1186/1475-9276-12-51 23837577 PMC3723953

[80] Ensor T Chhun C Kimsun T McPake B Edoka I. Impact of health financing policies in Cambodia: a 20 year experience. Soc Sci Med. (2017) 177:118–26. 10.1016/j.socscimed.2017.01.034 28161669

[81] Zaw PPT Htoo TS Pham NM Eggleston K. Disparities in health and health care in Myanmar. Lancet. (2015) 386:2053. 10.1016/S0140-6736(15)00987-3 26700385 PMC4672190

[82] Lagomarsino G Garabrant A Adyas A Muga R Otoo N. Moving towards universal health coverage: health insurance reforms in nine developing countries in Africa and Asia. Lancet. (2012) 380:933–43. 10.1016/S0140-6736(12)61147-7 22959390

[83] Su CW Huang SW Qin M. Umar M. Does crude oil price stimulate economic policy uncertainty in BRICS? Pac Basin Fin J. (2021) 66:101519. 10.1016/j.pacfin.2021.101519

[84] Paphassarang C Philavong K Boupha B Blas E. Equity, privatization and cost recovery in urban health care: the case of Lao PDR. Health Policy Plan. (2002) 17:72–84. 10.1093/heapol/17.suppl_1.72 12477744

[85] Tao R Su CW Xiao Y Dai K Khalid F. Robo advisors, algorithmic trading and investment management: wonders of fourth industrial revolution in financial markets. Technol Forecast Soc Change. (2021) 163:120421. 10.1016/j.techfore.2020.120421

[86] Kraipornsak P. Factors determining health expenditure in the Asian and the OECD countries. Econ World. (2017) 5:407–17. 10.17265/2328-7144/2017.05.003

[87] Su CW Qin M Tao R. Umar, M. Does oil price really matter for the wage arrears in Russia? Energy. (2020) 208:118350. 10.1016/j.energy.2020.118350

